# Maternal Microbiota, Cortisol Concentration, and Post-Partum Weight Recovery Are Dependent on Mode of Delivery

**DOI:** 10.3390/nu12061779

**Published:** 2020-06-15

**Authors:** Marta Selma-Royo, Izaskun García-Mantrana, Marta Calatayud, Anna Parra-Llorca, Cecilia Martínez-Costa, María Carmen Collado

**Affiliations:** 1 Department of Biotechnology, Institute of Agrochemistry and Food Technology (IATA-CSIC), Spanish Research Council, 46980 Valencia, Spain; mselma@iata.csic.es (M.S.-R.); igama@iata.csic.es (I.G.-M.); martacalatayud@yahoo.es (M.C.); 2 Neonatal Research Group, Health Research Institute La FE, University and Polytechnic Hospital La Fe, 46026 Valencia, Spain; annaparrallorca@gmail.com; 3 Department of Pediatrics, School of Medicine, University of Valencia, 46010 Valencia, Spain; cecilia.martinez@uv.es; 4 Pediatric Gastroenterology and Nutrition Section, Hospital Clínico Universitario Valencia, INCLIVA, 46010 Valencia, Spain

**Keywords:** microbiome, delivery mode, cortisol, post-partum weight retention

## Abstract

The importance of the maternal microbiota in terms of the initial bacterial seeding has previously been highlighted; however, little is currently known about the perinatal factors that could affect it. The aim of this study was to evaluate the effects of various delivery-related factors on the intestinal microbiome at delivery time and on post-partum weight retention. Data were collected from mothers (*n* = 167) during the first four months post-partum. A subset of 100 mothers were selected for the determination of the salivary cortisol concentration and microbiome composition at birth by 16S rRNA gene sequencing. The maternal microbiota was classified into two distinct clusters with significant differences in microbial composition and diversity. Maternal microbiota was also significantly influenced by the mode of delivery. Moreover, the salivary cortisol concentration was associated with some maternal microbiota genera and it was significantly higher in the vaginal delivery group (*p* = 0.003). The vaginal delivery group exhibited lower post-partum weight retention than the C-section (CS) mothers at four months post-partum (*p* < 0.001). These results support the hypothesis that the mode of delivery as well as the codominant hormonal changes could influence the maternal microbiota and possibly impact maternal weight recovery during the post-partum period.

## 1. Introduction

Gestation is a well-orchestrated process that affects all systems of the body, including the human microbiome [1,2]. The maintenance of pregnancy requires adaptations on the part of hormonal [3], immunological [4,5], and metabolic systems [6] in order to sustain fetal development. Several authors have compared these changes to those that occur in the case of metabolic syndrome, including insulin sensitivity [7] and an incremental increase in adiposity [8], which lead to sub-clinical inflammation [9] characterized by the increased production of proinflammatory cytokines. As the gut microbiota is interconnected with all these processes, its bacterial composition and diversity also undergo variations throughout pregnancy [1]. The variations seen in the microbiota during pregnancy tend toward an incremental increase in the presence of the Proteobacteria and Actinobacteria phyla as well as a reduction in diversity and the presence of some health-related genera [10]. In fact, the fecal transplantation of the gut microbiota from pregnant women during the third trimester of gestation to germ-free mice reproduced the symptoms of metabolic syndrome [10].

The maternal microbiota is considered to be the main driver of the initial bacterial seeding of the newborn [11]; however, the perinatal factors that affect the maternal microbiota are little understood, as prior studies have tended to focus on the factors shaping the infant microbiome [12,13]. Only a few studies have investigated the maternal microbiota and the elements that could modify its composition, including the mother’s diet during gestation [14,15,16,17,18], pregestational body mass index (BMI) [19,20], or weight gained during gestation [21]. In these studies, the importance of these factors, especially the maternal diet (e.g., fiber and fat-related nutrients), on intestinal microbiota at delivery was highlighted. Thus, little is currently known about the effects of the mode of delivery and associated factors on the composition of the maternal microbiome, while even less is known about their possible effects on the mother’s post-partum health status, which can reasonably be expected to affect neonatal development through mother–infant contact and breastmilk. 

The aim of this study was, therefore, to clarify the possible influence of factors related to the mode of delivery on the maternal microbiota and its association with maternal weight retention during the post-partum period.

## 2. Materials and Methods 

### 2.1. Participants and Sampling Information

Data from mother participants in the “impact of maternal microbes on infant health programming” (MAMI) cohort [22] were collected during the first four months post-partum for the present analysis (*n* = 167). Briefly, the MAMI cohort includes mothers and their offspring, who gave birth during the 2015–2018 period in the Valencia metropolitan area hospitals, including Hospital Universitario y Politécnico La Fe and Hospital Clínico Universitario. The exclusion criteria, objectives, and a description of the cohort are described in García-Mantrana et al. [22]. For the microbiota analysis, we selected a subset of 100 mothers from the MAMI cohort according to the availability of the sample at delivery. 

Anthropometric and clinical data from those mothers were collected, including the delivery mode, maternal age, pre-pregnancy BMI, antibiotic intake during pregnancy, or weight gain during gestation, among others. Delivery-related data, such as rupture of membranes mode, instrumentalization, and antibiotic administration, were also recorded. 

The dietary intake of the mothers was evaluated by a 140-item validated food questionnaire (FFQ) [23] that mothers filled within the first week after delivery. FFQ information was analyzed using the Nutrients Food Composition Tables developed by the Centro de Enseñanza Superior de Nutrición Humana y Dietética (CESNID), Spain [24].

All mothers received oral and written information about the study and all participants provided written informed consent before enrolment. Ethical approval for the study was obtained from the Ethics/Bioethics Committee for Clinical Research of Hospital Universitario y Politécnico La Fe, Hospital Clinico Universitario and CSIC (Consejo Superior de Investigaciones Científicas) [ClinicalTrial.gov NCT03552939].

### 2.2. Biological Samples

Fecal samples at delivery were collected using a sterile cotton-tipped probe by trained clinical staff before birth in the delivery room following the previously described protocol [22]. Similarly, saliva samples were also collected in special sponge dispositive devices (Salivette, Sarstedt AG & Co. KG, Nümbrecht, Germany) in the delivery room. All the C-section mothers, both ECS and CS, received intravenous intrapartum antibiotics during the intervention. Samples were collected before the surgery was initiated. In the case of emergency C-section, the labor process was begun, but it was not completed due to complications, including abnormal neonatal position, neonatal size, problems in heart rate, blood pressure, etc. In the case of mothers who underwent a vaginal delivery, all of them had the labor initiated and the samples were collected during the dilatation time. 

All samples were sent to biobank, and then, biological samples were managed and stored at −80 °C in sterile cryovials under specific standardized protocols at “Biobanco para la Investigación Biomédica y en Salud Pública de la Comunidad Valenciana (IBSP-CV)”. Once all cohort samples were collected and placed in the biobank, aliquots were shipped and centralized at Institute of Agrochemistry and Food Technology (IATA-CSIC) for the analysis.

### 2.3. Cortisol Concentration Quantification 

Saliva was collected from the Salivette device by centrifugation of the sponge contained in the tube. The resulting liquid was transferred to a new tube and stored at −80°C until analysis. The saliva cortisol concentration was assessed by the immunoassay kit “Salivary Cortisol ELISA KIT” (Salimentrics Assay #1-3002, Salimetrics, Carlsbad, CA, USA) following the manufacturer’s instructions. 

### 2.4. DNA Extraction

DNA was extracted from the fecal material using the Master-Pure DNA extraction kit (Epicentre, Madison, WI, USA) following the manufacturer’s instructions. Some additional steps were added to the basic protocol, including a treatment for 60 min at 37 °C with lysozyme (20 mg/mL) and mutanloysin (5 U/mL) followed by two cycles of cell disruption with 3-μm-diameter glass beads performed by a bead beater FastPrep 24-5G Homogenizer (MP Biomedicals) during 30 s at 6 m/s. Then, purification of the DNA was assessed by the DNA Purification Kit (Macherey-Nagel, Duren, Germany) according to the manufacturer’s instructions and the resulting DNA concentration was measured using a Qubit^®^ 2.0 Fluorometer (Life Technology, Carlsbad, CA, USA). 

### 2.5. Amplicon Sequencing and Bioinformatics

The amplification of the V3–V4 variable region of the 16S rRNA gene was conducted following the 16SrDNA gene Metagenomic Sequencing Lybrary Preparation Illumina protocol (Cod. 15044223 Rev. A) with the primers selected from [25]. A NextERA XT Index Kit (FC-131-2001) (Illumina, San Diego, CA, USA) was used for the multiplexing step, and the DNA quality of the resulting library PCR product was assessed by a Bioanalyzer DNA 1000 chip (Agilent Technologies, Santa Clara, CA, USA). Then, libraries were sequenced using a 2 × 300 bp paired-end run (MiSeq Reagent kit v3) on a MiSeq-Illumina platform (FISABIO sequencing service, Valencia, Spain) according to the manufacturer’s instructions. The obtained reads were searched for residual adaptors using the program Trimmomatic [26]. 

The DADA2 pipeline [27] was followed for the quality trimming and filtering of the obtained sequences. Reads were trimmed at the 270th and 210th nucleotide in the forward and reverse position, respectively, after quality examination. Additionally, adapters were also removed in the filtering process and a maximum of 2 expected errors was considered. Then, the sequences were merged, and chimera removed following the mentioned pipeline with the default options. The Silva v132 database [28] was used for the taxonomic assignment with the species-level classification. 

Additional filters were also performed as follows: Taxa occurring <3 reads in at least 20% of the samples, and those that represented less than 0.01% of the total reads across all the samples were also removed. Furthermore, the decontam package [29] in R environment [30,31] was used to determine the presence of potential contaminant-related sequence. Samples with less than 1000 reads were also removed from the final analysis and bacterial counts were transformed to the relative abundance.

### 2.6. Statistical Analysis

Differences in population anthropometric and clinical data was tested by *t*-test and Mann–Whitney analysis according to the data normality assessed by the Shapiro-Wilk test in Graphpad software v. 5.04 (GraphPad Software, La Jolla, CA, USA, www.graphpad.com). Chi-squared test (2 × 2) was performed to assess the significance of the differences in the characteristics according to categorical variables. Mothers were classified according their pregestational BMI [32] and their weight gain during pregnancy category following the recommendations of the Institute of Medicine [33] for each pre-gestational BMI. 

Maternal microbiota clustering was generated at the genus level as described elsewhere [34] using the phyloseq [35], cluster [36], MASS [37], clusterSim [38], and ade4 R packages [39]. Briefly, the Jensen–Shannon distance and partitioning around medoid (PAM) clustering were used. The optimal number of clusters was calculated by the Calinski-Harabasz (CH) index. 

RStudio (R v. 3.6.1) was used to perform the PERMANOVA (Adonis) multivariate analysis in maternal and neonatal microbiota by the vegan package [40] based on the Bray-Curtis distance. Vegan package was also used for the alpha diversity by Shannon (diversity estrimation) and Chao1 indexes (richness estimation), and the Kruskal-Wallis/Mann-Whitney test with false discovery rate (FDR) adjustment was performed to test significance between variables. Partial Spearman correlation adjusted by BMI was used to assess the relation between weight gain over pregnancy and maternal microbiota at delivery. 

Discriminant analysis of principal components (DAPC) and the Adonis test were also achieved based on the Bray-Curtis distance in the Calypso online platform v. 8.84 [41]. Boxplots showing differences in microbial genera were generated in GraphPad software with the log10 transformation to facilitate visualization. The Calypso online platform was also used to test significant differences in the microbiota composition according to the studied variables. All comparisons were adjusted by FDR. 

Spearman correlations in both delivery modes were conducted in SPSS v. 25 (IBM, Armonk, NY, USA) [42] software to describe the association between the salivary cortisol concentration and maternal microbiota genera. A multivariate model adjusted for pre-pregestational BMI, weight gain during pregnancy, and breastfeeding duration was performed on maternal weight variations according to the delivery mode during the post-partum period using SPSS v.25. The maternal post-partum weight recovery (PPWR) evolution was studied based on the maternal weight (kg) for 4 months post-partum, and the difference with the pre-gestational weight was also calculated. In the weight recovery analysis, mothers were considered to recover the pre-gestational weight when they had a difference between the pre- and post-partum weight below the mean difference + 1SD (standard deviation).

## 3. Results

### 3.1. Study Participants’ Characteristics

The characteristics of the participating mothers in the microbiota study presented a median age of 35 years old and showed a pre-gestational body mass index (BMI) of 22.58 kg/m^2^, which was within the normal weight range (Table 1). 

In total, 54.6% of the mothers underwent a vaginal labor (VAG) while 36.1% had an elective C-section (CS) and the rest (9.3%) had an emergency C-section (ECS). VAG and ECS pregnancies showed a slightly higher gestational age than elective C-section (*p* < 0.001). Besides this, no differences were found in any of the other studied factors between the three groups. Furthermore, no differences in nutrient intake were found between the maternal groups according to the delivery mode (Table 1). The characteristics of the population used in the study of the effect of the delivery mode on the postpartum weight retention (PPWR) are listed in Supplementary Material Data (Supplementary Table S1). 

### 3.2. Maternal Microbiota Composition at Delivery Time

Maternal microbiota at delivery were characterized by a dominance of Firmicutes and Bacteroidetes phyla, with relative abundances of 68.07% and 18.59%, respectively, followed by Actinobacteria (5.75%) and Proteobacteria (3.50%) phyla (Supplementary Material, Figure S1). 

**Figure 1:**
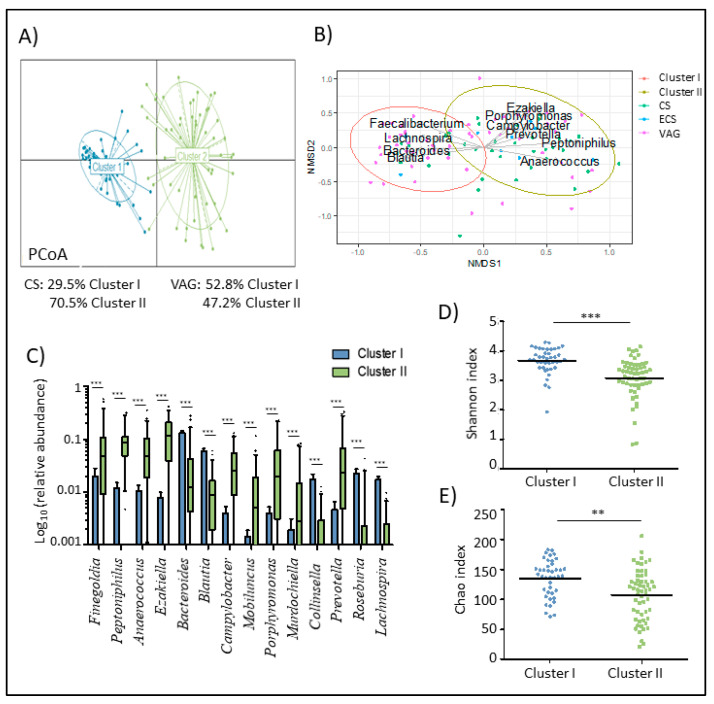
Maternal microbiota was clustered in two groups based in their composition and diversity at delivery. (**A**) Principal coordinate analysis (PCA) of maternal microbiota at delivery according to the cluster at the genus level. (**B**) Non-metric multidimensional scaling (NMDS) of the maternal microbiota at delivery time at amplicon sequence variant (ASV). Arrows show the genus loadings for each cluster. the color of the points expresses the delivery mode group of the mother. (**C**) Boxplot of the main genera that marked the difference of the maternal microbiota composition between both clusters. Data was transformed by log10 of the relative abundance of each genus for plotting. Whiskers represent the 5-95 percentile interval. (**D**,**E**) Differences in the diversity (**D**) and richness (**E**) of maternal microbiota based on the Shannon index according to the cluster. Line represents the median of each group. * *p* < 0.05. ** *p* < 0.01. *** *p* < 0.001.

We performed a clustering of mothers enrolled in the study according to their microbiota at delivery. The results revealed two distinct microbial clusters (Figure 1A,B). Cluster I was characterized by higher relative abundances of *Blautia, Lachnospira, Bacteroides, and Faecalibacterium* genera. However, Cluster II was predominantly dominated by *Ezakiella*, *Peptoniphilus*, *Campylobacter*, and *Porphyromonas* genera (Figure 1C).

A higher relative abundance of *Blautia* (*p* < 0.001), *Rombustia* (*p* < 0.001), *Roseburia* (*p* < 0.001), *Lachnospira* (*p* < 0.001), and several groups from the Ruminococcaceae family was found in cluster I compared to cluster II. However, cluster II mothers had a higher relative abundance of *Prevotella* (*p* < 0.001), *Mobiluncus* (*p* < 0.001), *Lawsonella* (*p* < 0.001), *Murdochiella* (*p* < 0.001), *Corynebacterium* (*p =* 0.001), and *Lactobacillus* (*p* = 0.003), among others.

At the phylum level, only the Epsilonbacteraeota (*p* < 0.001) phylum, which represents less than 1% of the total reads, showed significant differences between clusters (Supplementary Figure S1). Furthermore, differences in the relative abundance of Euryarchaeota (*p* = 0.015) were also found between clusters. 

In terms of the alpha-diversity (Figure 1D,E), mothers classified in cluster I showed higher microbial diversity and richness measured as the Shannon (*p* < 0.001) and Chao1 (*p* = 0.001) index, respectively.

### 3.3. Factors Affecting Maternal Microbiota at Delivery

**Figure 2:**
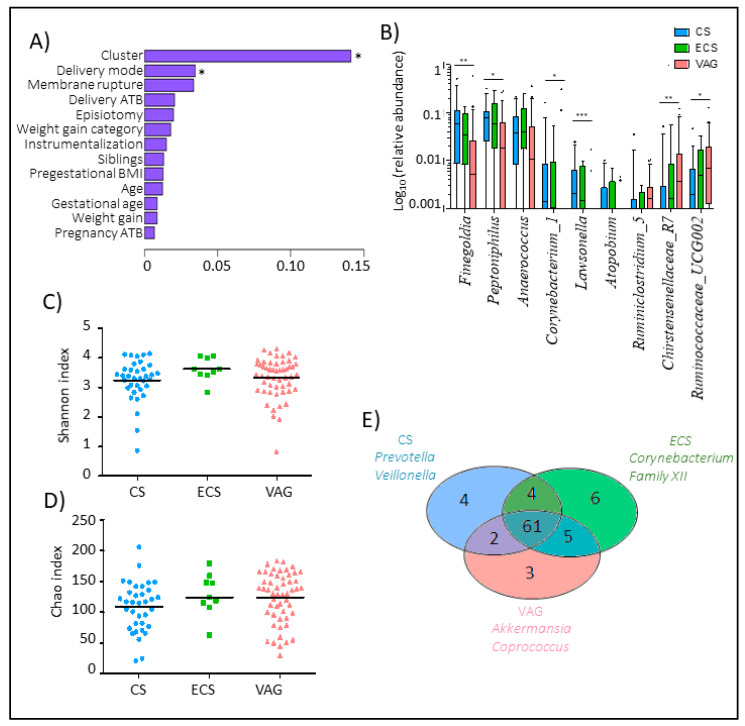
Maternal microbiota composition is influenced by the delivery mode. (**A**) Multivariate analysis of the effect of the studied perinatal factors on the maternal microbiota composition at delivery based on Bray-Curtis. Membrane rupture. antibiotic at delivery, and episiotomy were only studied in mothers who had vaginal delivery. (**B**) Boxplot of the main genera that marked the difference of the maternal microbiota composition between delivery modes. Data was transformed by log10 of the relative abundance of each genus for plotting. Whiskers represent the 5–95 percentile interval. (**C**) The core group of the maternal microbiota composition at the genus level performed by the Venn diagram. (**D**,**E**) Differences in the diversity (**D**) and richness (**E**) of maternal microbiota based on the Shannon index according to the delivery mode. The line represents the median of each group. CS (Elective C-section). ECS (Emergency C-section). VAG (Vaginal delivery). * *p* < 0.05. ** *p* < 0.01. *** *p* < 0.001.

We performed a permutational multivariate analysis of variance (PERMANOVA) based on the Bray-Curtis distance to clarify the relevance of the perinatal factors and maternal microbiota composition at delivery (Figure 2A). Delivery mode (*p* = 0.003) significantly affected the maternal microbiota composition at delivery followed by the rupture of membranes mode (*p* = 0.07), which was only analyzed in vaginal deliveries since all CS were carried out during the intervention. 

At the genus level (Figure 2B) (Supplementary Figure S2), CS women showed a higher relative abundance of *Lawsonella* (*p* < 0.001), *Finegoldia* (*p* = 0.001), and *Corynebacterium* (*p* = 0.004) genera, among others, compared to VAG deliveries. However, women with VAG showed an enrichment in Christensenellaceae_R7_group (*p* = 0.002) and some groups from the Ruminococcaceae groups, including Ruminococcaceae_UCG002 (*p* = 0.053), Ruminococcaceae_UCG009 (*p* = 0.053), and *Ruminiclostridium_*5 (*p* = 0.053). While we found significant differences in the microbiome composition between VAG and CS mothers, no differences were found between VAG and ECS mothers. Moreover, no differences in terms of the alpha-diversity were observed between the three studied delivery modes, neither in the diversity nor richness index (Figure 2C,D).

We found a microbial core in which the genera prevalence was not affected by the delivery mode that included several groups from the Ruminococcaceae family, *Romboutsia*, *Roseburia*, *Prevotella*, and *Faecalibacterium* genera, among others (Supplementary Table S2). Venn diagrams revealed that ECS and VAG shared a higher number of genera than elective CS (Figure 2E). Mothers that had an emergency CS were characterized by a prevalence of *Corynebacterium* and Clostridiales_Family XII while those that had an elective CS were characterized by a higher prevalence of *Prevotella* and *Veillonella* genera. Microbiota from VAG mothers were overrepresented by *Akkermansia* and *Coprococcus* genera. 

**Figure 3:**
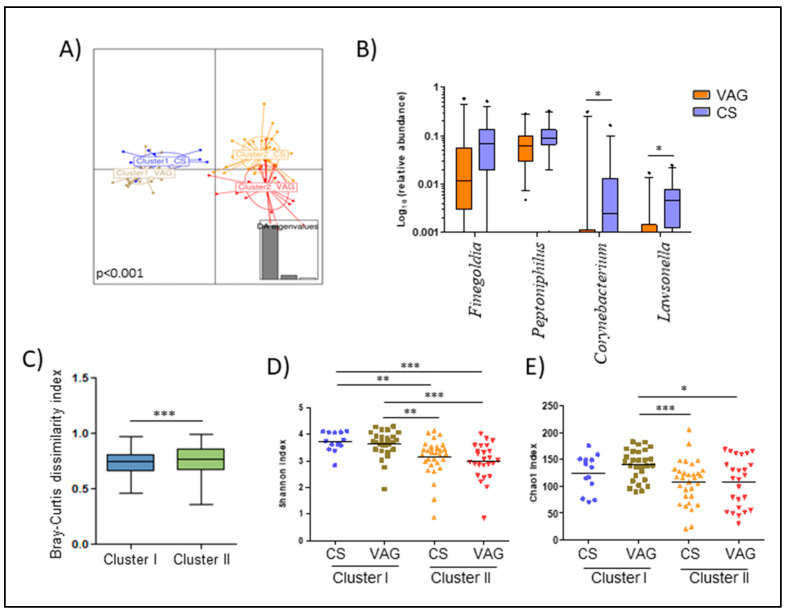
Maternal microbiota at delivery is dissentingly shaped by the delivery mode. (**A**) Discriminant analysis of principal components (DAPCs) of the maternal microbiota according to the variable that resulted from the combination of the cluster and delivery mode. (**B**) Boxplot of the main genera that marked the difference of the maternal microbiota composition between delivery modes in mothers classified as cluster II. Data was transformed by log10 of the relative abundance of each genus for plotting. Whiskers represent the 5–95 percentile interval. (**C**) Bray-Curtis dissimilarity index within mothers that had both vaginal and C-section delivery according to the cluster classification. (**D**,**E**) Differences in the maternal microbiota diversity (**D**) and richness (**E**) based on the Shannon and Chao1 index, respectively, according to the variable that resulted from the combination of the cluster and delivery mode. The line represents the median of each group. * *p* < 0.05. ** *p* < 0.01. *** *p* < 0.001.

While mothers who gave birth by VAG were equally distributed in both clusters, cluster II was enriched in mothers that had a CS (55.4% prevalence in cluster II vs. 31.7% in Cluster I). Since the results suggested this could have an interference between both factors, we decided to study their possible effect on maternal microbiota, independently. We observed that mothers classified in cluster II were more sensitive to alterations related to the delivery mode (*p* < 0.001) (Figure 3A,B). Indeed, the Bray-Curtis dissimilarity index showed that mothers from cluster I had a microbiota more similar between both delivery modes than those mothers from cluster II (*p* = 0.004) (Figure 3C). Within cluster II, CS mothers showed a higher relative abundance of *Lawsonella* (*p* = 0.014) and *Finegoldia* (*p* = 0.082) while VAG mothers were enriched in *Corynebacterium* genera (*p* = 0.029) (Figure 3B). 

In terms of alpha-diversity (Figure 3D,E) and within VAG mothers, those belonging to cluster I had higher richness than both groups of mothers in cluster II, those from CS (*p* = 0.009) and vaginal birth (*p* = 0.017). Additionally, mothers from cluster I from both the CS and VAG groups showed higher diversity than those from cluster II (*p* < 0.01) (Figure 3D). 

Regardless of the other studied factors, we found significant relations between weight gain over pregnancy and maternal microbiota at delivery. Weight gain during pregnancy was negatively associated with the *Escherichia/Shigella* genus (ρ = −0.51, *p* < 0.001) from Proteobacteria phylum (ρ = −0.46, *p* < 0.001) as well as Christensenellaceae_R7_group (ρ = −0.30, *p* = 0.023). No significant differences were found among VAG mothers according to antibiotic administration at delivery. 

### 3.4. Delivery Mode Was Related to Saliva Cortisol Concentrations

**Figure 4:**
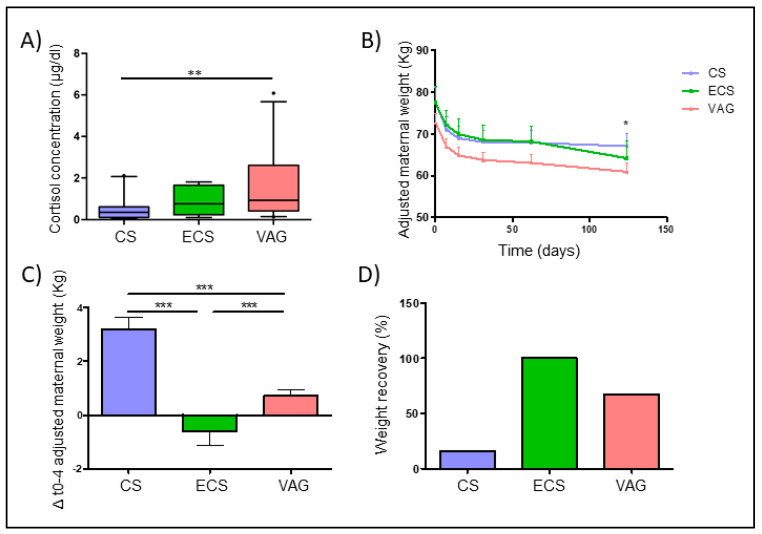
Delivery mode affected the saliva cortisol concentration and weight gain recovery four months post-partum. (**A**) Differences in the saliva cortisol concentration (µg/dL) according to the delivery mode. (**B**) Evolution of the adjusted maternal weight by breastfeeding during this time, pre-gestational weight, and weight gain over pregnancy from delivery to 4 months post-partum. Data were expressed as mean and 95% CI. (**C**) Differences in the increment of the maternal weight from pre-gestational weight to 4 months post-partum. (**D**) Percentage of mothers that had a recovery of their pre-gestational weight 4 months post-partum. Mother was considered to have recovered her pre-gestational weight if they showed a difference between the pre- and post-partum weight below the mean difference + 1SD (standard deviation). * *p* < 0.05. ** *p* < 0.01. *** *p* < 0.001.

The cortisol concentration in maternal saliva at delivery was 0.55 [IQR: 0.24–1.26] µg/dL. Significant differences were found in the saliva cortisol concentration according to the delivery mode (*p* = 0.003) (Table 1). While ECS mothers showed no differences in terms of the cortisol concentration with VAG mothers, those from the elective CS group presented lower cortisol concentrations compared to VAG mothers (*p* = 0.001) (Figure 4A). 

Furthermore, the saliva cortisol concentration was related to the maternal microbiota composition (Table 2). Saliva cortisol was negatively associated to *Finegoldia* (ρ = −0.43, *p* = 0.024), *Peptoniphilus* (ρ = −0.36, *p* = 0.062), *Corynebacterium* (ρ = −0.66, *p* < 0.001), and *Lawsonella* (ρ = −0.54, *p* = 0.003) independently of the delivery mode effect. Indeed, the salivary cortisol concentration showed a positive correlation with Christensenellaceae_R7_group (ρ = 0.37, *p* = 0.033) and genera from the Lachnospiraceae and Ruminococcaceae groups, including *Ruminococcus_1* (ρ = 0.42, *p* = 0.031) and *Lachnoclostridium* (ρ = 0.44, *p* = 0.023), and Lachnospiraceae_UCG_010 (ρ = 0.41, *p* = 0.017). 

### 3.5. Delivery Mode with Post-Partum Maternal Weight Retention

Multivariate analysis adjusted by covariates, including pre-gestational BMI, weight gain during pregnancy, and breastfeeding duration, showed a significant effect of the delivery mode on weight loss over the 4 months post-partum (Figure 4B) (*p* < 0.001). Mothers who gave birth vaginally showed a lower difference between their weight at 4 months post-partum and their pre-gestational weight, compared to CS group mothers (*p* < 0.001) (Figure 4C). Indeed, a higher number of mothers who recovered their pre-gestational weight status were found within VAG mothers (67.28%) than in the group of CS mothers (16.13%) (Figure 4D). 

## 4. Discussion

Pregnancy is an extremely demanding process that affects all the systems of the body, including the maternal microbiota [1]. Several authors have described the variations that occur during gestation, mainly in the oral [43], vaginal [44], and intestinal microbiomes [45]. Furthermore, this period is recognized as a unique time in which health care designed to improve the mother’s well-being can affect both maternal and neonatal health. Thus, interventions during pregnancy may provide an opportunity to positively impact the long-term health of the mother and her offspring. The present study aimed to describe the effects of various perinatal factors on the maternal microbiome at the time of delivery as well as its associations with post-partum maternal weight retention. 

Several prior studies have highlighted the importance of the maternal microbiome in relation to the initial colonization of the neonatal microbiota [11,17,18,46,47]. However, only a few studies have evaluated the effects of pregnancy and delivery-related factors on maternal physiology and body composition after birth. In particular, the influence of the metabolic, immunological, and hormonal shifts that occur during delivery on the maternal microbiota are only poorly understood. It is important to recognize that birth represents the first contact between the newborn and a significant bacterial challenge, and further, that the maternal microbiota determines the nature of that initial bacterial exposure [48,49]. 

We identified two bacterial patterns in the maternal microbiota composition and richness at the time of delivery. One of the clusters (cluster I) was composed of mothers with a higher microbial richness and diversity, and a microbiota characterized by the presence of health-related bacteria, such as *Blautia*, *Roseburia*, *Lachnospira*, and *Faecalibacterium* genera. The other group of mothers (cluster II) presented less rich and less diverse microbiota, which were dominated by *Finegoldia*, *Peptoniphilus*, *Campylobacter*, *Prevotella*, *Porphyromonas*, and *Lawsonella*, among others. 

The bacteria that dominated in cluster I are commonly related to the health status of the non-pregnant human microbiota [50,51], mainly due to their ability to produce short-chain fatty acids (SCFAs) [51], which is derived from their capacity to metabolize certain dietary compounds, including fiber [52,53]. Propionate, acetate, and butyrate are all associated with multiple functions in the gut and other host systems [54], and they are considered to be the metabolites that control the interplay between the diet, microbiota, and host metabolism [55]. Indeed, these molecules, which are derived from the maternal microbiota, are also known to be associated with metabolic parameters in neonates [56], even participating in immune system development [57]. Additionally, in the MAMI cohort, García-Mantrana et al. [17] identified an association between a cluster represented by the same health-related genera and a higher intake of plant-derived components, including total dietary fiber, omega-3 fatty acids, and polyphenols. Thus, the gut microbial profiles observed at the time of delivery could be partially modulated by the maternal diet during gestation.

Although several prior studies have demonstrated the effect of the mode of delivery on the neonatal microbiota at the time of birth and thereafter [58], little is currently known about the effect of the mode of birth on the maternal microbiota. In the present study, we found that the mode of delivery significantly affected the composition of the maternal microbiota. More specifically, CS delivery was found to be associated with higher relative abundances of *Corynebacterium* and *Lawsonella* genera as well as the Gram-positive anaerobic cocci (GPAC), including *Finegoldia* and *Peptoniphilus* genera, while VAG delivery was associated with higher abundances of the *Christensenellaceae* and *Ruminococcaceae* families. Interestingly, the mothers who underwent an emergency CS (ECS) showed an intermediate pattern regarding the microbial composition of their microbiota, which was somewhere between the patterns associated with VAG delivery and elective CS delivery. It is important to recognize that an ECS is normally performed after the hormonal and metabolic processes associated with delivery have been triggered [59,60]. In addition, mothers who undergo an ECS do not usually follow the practice of pre-fasting, which is required for a scheduled CS [61,62]. Thus, our results are in accordance with the hypothesis that hormonal and clinical differences related to the mode of delivery could induce shifts in the maternal gut microbiome, which could have consequences in relation to mother–infant transference during delivery. 

Although prior research has focused on just a few factors, such as the use of antibiotics or the delivery mode, the host-microbiota interactions during the perinatal period could be more complex than initially thought, which means that other potential factors, such as diet or hormonal status, should be considered. In the present study, the mothers in cluster II, which was characterized by less diversity, presented higher rates of CS delivery. However, the VAG delivery mothers were equally distributed among both clusters. Indeed, while the cluster I mothers had microbiota that were less influenced by the mode of delivery, the identified birth-related shifts in the gut microbiota were more pronounced in the cluster II mothers. 

Several authors have proposed diversity, stability, and resilience to be characteristics associated with a healthy microbiota [63,64]. A resilient ecosystem is capable of remaining stable and resistant in relation to perturbations over time without losing its equilibrium [65]. Both species and functional diversity are the principal features that promote the resilience of the bacterial community. High diversity would allow minor species to fill a niche if the more abundant species were diminished by a disturbance. In the present study, the mothers from cluster I, which was characterized by richer and more diverse microbiota, also presented more robust microbiota, which led to less shifts being induced by the delivery mode when compared with those seen in cluster II. This probably reflects the higher stability and resilience of their gut microbial communities. 

The triggering of labor induces several biological processes, including hormonal and immunological system responses, which culminate in childbirth [59,60]. In this study, the saliva cortisol concentration was found to be increased in the mothers who underwent VAG delivery. Cortisol determination has previously been proposed as a stress marker in several studies [66] involving pregnant populations [67]. Further, pregnancy has been suggested to be associated with an increased cortisol concentration throughout the gestation period [68,69]. Indeed, labor is widely assumed to be a stressful process. Miller et al. found the salivary cortisol concentration to gradually increase from the latent to the active phase in low-risk VAG deliveries, with the maximum concentration being observed within two minutes of the onset of labor [70]. In line with our results, some authors have described how VAG deliveries are associated with higher serum cortisol concentrations and more intense inflammation than elective CS deliveries, although the inflammatory response period is shorter than in the case of CS [71,72,73]. 

Aside from cortisol, other bioactive molecules have been observed to a greater extent in the maternal plasma obtained from VAG deliveries than in that obtained from CS deliveries [74]. Despite evidence suggesting that the maternal host systems could interact with and alter the human microbiota [75], the possible effects of the physiological response that occurs during labor, as well as during the immediate pre- and post-labor periods, have not yet been adequately investigated. Our results suggest a possible relation between the salivary cortisol concentration and the composition of the maternal gut microbiota at delivery. Even in mothers who have undergone a VAG delivery, the cortisol concentration has been found to be negatively related to those genera associated with CS delivery, including the above-mentioned GPAC (*Finegoldia*, *Peptoniphilus*, and *Anaerococcus*). However, the genera known to be enriched in VAG delivery mothers, including genera from the *Christensenellaceae* and *Ruminococcaceae* families, were observed to be positively associated with the salivary cortisol concentration. These results suggest that the cortisol concentration may play a role in the delivery-mode-related shifts observed in the maternal microbiota. The possible mechanisms by which the circulating cortisol concentration could modify the maternal gut composition include bile acid and cholesterol regulation, acidic gut secretion and motility alterations, and gut barrier function disruption [76]. Other perinatal labor-related physiological changes could also influence the observed clustering of mothers based on their gut microbiota. 

Furthermore, both VAG delivery and the cortisol concentration are known to be associated with health-related genera, including members of the *Christensenellaceae* [77], *Lachnospiraceae*, and *Ruminococcaceae* families [51]. In fact, Vojinovic et al. found these groups to be correlated and involved in several metabolic pathways, including SCFA production and bile acid metabolism [78]. In particular, the *Christensenellaceae* family has been observed to be inversely associated with the BMI [79,80] of lean individuals [81], as well as with a healthy condition, in studies investigating inflammatory bowel disease (IBD) [82] and other inflammatory gut-related disorders [83,84]. For instance, Papa et al. reported lower levels of *Christensenellaceae* in fecal samples obtained from pediatric and young adult intestinal bowel disease (IBD) patients when compared with samples obtained from healthy controls [85]. In the present study, we identified a negative correlation between weight gain during pregnancy and this bacterial group. Furthermore, some prior studies have suggested that this family could be among the most hereditable families [80,86], which suggests that it might exert an influence on neonatal health. 

In this study, more VAG delivery mothers were found to have returned to their pre-pregnancy weight after four months post-partum than CS delivery mothers. Post-partum weight retention (PPWR) has been identified as an important challenge facing public health systems worldwide due to its negative association with both maternal recovery and neonatal development [87]. The use of CS is commonly associated with longer recovery times, a special post-surgical intervention diet [88,89], and delayed breastfeeding [90,91], which could also influence PPWR. 

The mother’s pregestational BMI and gestational weight gain have been found to be related to increased PPWR during the post-partum period [92]. Our model, when adjusted according to these covariables, indicated that VAG delivery mothers exhibit significantly lower PPWR. However, the mother’s pregestational BMI and gestational weight gain have also been proposed as risk factors in relation to CS intervention [93]. Thus, although several epidemiological studies have identified higher PPWR in mothers who have undergone CS deliveries, there remain contradictions in terms of the overall results [94], possibly due to the complex interactions that occur between all these factors. 

As mentioned above, metabolites other than cortisol may have influenced the delivery-mode-related differences we observed regarding both the microbiota composition and post-partum weight loss. Recently, Koren et al. demonstrated that the progesterone concentration could influence the relative abundance of *Bifidobacterium* in the maternal gut microbiota [95]. Further, Rebelo et al. identified differences in the adiponectin concentration according to the mode of birth, with CS delivery being associated with a lower adiponectin concentration than VAG delivery at 30–45 days post-partum, which was likely mediated by the long-term inflammation associated with the CS procedure [96]. 

It is important to recognize that the present study had a few limitations, including the low sample size, which might have influenced the power analysis of the observed results. Additionally, the lack of a microbiota profile during the post-partum period represents another weakness of the study. However, the main aim of the analysis was to describe the effects of delivery-related factors on the maternal microbiota at the time of birth. Among the strengths of this study, we wish to highlight the detailed information gathered regarding the maternal clinical and anthropometrical characteristics, which allowed us to evaluate multiple factors within the same population. Our contact with hospitals and community health centers greatly helped in reducing the rate of data collection mistakes and also decreased the amount of missing data. 

## 5. Conclusions

In summary, the results of this study suggest the existence of a complex relation between hormonal delivery-related changes, the gut microbiota, and maternal health outcomes in the post-partum period, which might impact on neonatal development during this important period and thus have long-term consequences. However, the link between these factors has been underexplored in the prior maternal health research. Our results support the hypothesis that the mode of delivery and codominant hormonal changes could influence the maternal microbiota at the point of delivery. Further research is required to comprehensively explain the molecular mechanisms that mediate the host–microbiota interplay at birth in order to design clinical strategies for improving maternal and neonatal health during the post-partum period and beyond.

## Figures and Tables

**Table 1:** Characteristics of the population in the microbiota study (*n* = 97).

	Total (*n* = 97)	Vaginal (*n* = 53)	Emergency C-Section (*n* = 9)	Elective C-Section (*n* = 35)	*p*
**Pregnancy data**					
Maternal Age (years)	35 (31–37)	33 (29.50–36.50)	35 (32.50–36)	35 (31–38)	0.200
Weight Gain (kg)	12 (10–15)	12 (10–14)	15 (9.85–20)	12.5 (10.5–15.30)	0.242
Low	21 (21.7)	16 (30.2)	3 (33.3)	6 (17.1)	<0.001 *
Recommended	51 (52.6)	30 (56.5)	2 (22.2)	19 (54.3)	
High	25 (25.8)	7 (13.2)	4 (44.4)	10 (28.6)	
Pre-gestational BMI (kg/m^2^)	22.58 (20.43–25)	21.63 (20.14–24.06)	20.32 (19.42–23.49)	23.42 (21.01–25.88)	0.070
NW	68 (70.1)	39 (73.6)	7 (77.8)	22 (62.9)	0.001 *
OW	23 (23.7)	10 (18.9)	1 (11.1)	12 (34.3)	
LW	6 (6.2)	4 (7.5)	1 (11.1)	1 (2.9)	
Antibiotic at Pregnancy	40 (41.2)	23 (43.4)	4 (44.4)	13 (37.1)	0.525
Gestational Age (weeks)	39 (38–40)	40 (39,40) b	39 (38–40) ab	38 (38–40) a	<0.001 *
Siblings	35 (36.1)	18 (34)	1 (11.1)	16 (45.7)	<0.001 *
**Delivery data**					
O’Sullivan index	124.7 ± 28.78	122.5 ± 27.668	127.1 ± 33.87	121.5 (107–154.3) 7	0.821
Salivary Cortisol (µg/dL)	0.55 (0.23–1.32)	0.93 (0.42–2.61)26 b	0.77 (0.23–1.65)2 ab	0.35 (0.11–0.61) 9 a	0.003 *
Intrapartum Antibiotic	7 (13.2)	7 (13.2)	9 (100)	35 (100)	-
Episiotomy	18 (34) 8	18 (34) 8	-	-	-
Instrumentalization	12 (22.7)	12 (22.7)	-	-	-
Rupture of Membranes					
Spontaneous	33 (62.3)3	33 (62.3)3	-	-	-
Artificial	17 (32.1)	17 (32.1)	-	-	-
***Dietary data***					
Energy (Kcal)	2770 (220–3275)	2817 (2313–3382)	3040 (2103–4157)	2309 (2115–3051)	0.092
Total Proteins (g)	113 (94–130)	116 (94–127)	107 (97–162)	107 (87–103)	0.485
Animal Proteins (g)	74 (58–89)	73 (57–87)	75 (64–103)	74 (55–96)	0.690
Vegetable Proteins (g)	39 (30–49)	41 (33–51)	32 (28–73)	32 (25–29)	0.077
Lipids (g)	137 (111–170)	142 (111–171)	149 (101–205)	133 (111–148)	0.380
SFA (g)	28 (28–49)	38 (27–49)	42 (27–58)	38 (28–49)	0.902
MUFA (g)	69 (52–80)	71 (56–80)	79 (54–95)	68 (47–74)	0.174
PUFA (g)	22 (17–30)	23 (18–29)	18 (13–39)	22 (17–30)	0.986
Cholesterol (mg)	390 (332–482)	403 (331–486)	393 (357–508)	370 (305–465)	0.643
Carbohydrates (g)	264 (205–346)	287 (222–353)	249 (76–364)	210 (160–288)	0.062
Fiber (mg)	32 (23–45)	39 (25–47)	34 (29–53)	26 (21–39)	0.122

Significance differences were tested in the three studied groups and were marked in the table with an asterisk (*). Normally distributed data was presented as mean ± SD and non-normal data as median [IQR]. Categorical variables were expressed as positive cases (percentage). Data no sharing letters was significantly different between studied groups. Number of missing values for each variable was represented as a superscript. NW (Normoweight), OW (Overweight), LW (Low weight).

**Table 2:** Spearman correlation between the salivary cortisol concentration and maternal microbiota at delivery.

Genus	ρ	*p*-Value	Rel. Abund.
*Peptoniphilus*	−0.36	0.062	3.47 (0.49–9.04)
*Finegoldia*	−0.43	0.024	2.9 (0.14–13.42)
*Lachnoclostridium*	0.44	0.023	0.27 (0.05–0.72)
*Christensenellaceae_R_7_group*	0.37	0.033	0.23 (0.01–0.90)
*Corynebacterium_1*	−0.66	<0.001	0.17 (0–0.27)
*Lawsonella*	−0.54	0.003	0.06 (0–0.28)
*Staphylococcus*	−0.60	0.001	0 (0–0.42)
*Lachnospiraceae_UCG_010*	0.41	0.017	0 (0–0.13)
*Ruminococcus_1*	0.42	0.031	0 (0–0.10)
*Arcanobacterium*	−0.47	0.013	0 (0–0.02)

Spearman correlations were performed separately in vaginal or C-section deliveries in order to avoid its effect on both cortisol concentrations and maternal microbiota. Relative abundance was expressed as the median of percentage (interquartile range) of total reads obtained for each fecal sample.

## Supplementary Materials

The following are available online at https://www.mdpi.com/2072-6643/12/6/1779/s1: Figure S1: Maternal microbiota composition at phylum level at delivery time. Figure S2: Differences in maternal microbiota at delivery time according to delivery mode. Table S1. Characteristics of population participant in the post-partum weight retention study (*n* = 167). Table S2: Occurrence of genera from maternal microbial core at delivery according to delivery mode.

## Abbreviations

VAG	Vaginal delivery
CS	Elective C-section
ECS	Emergence C-section
BMI	Body Mass Index
PPWR	Post-Partum Weight Retention
SCFA	Short-Chain Fatty Acids
GPAC	Gram Positive Anaerobic Cocci
