# Supplementary material for: Maternal Microbiota, Cortisol Concentration, and Post-Partum Weight Recovery Are Dependent on Mode of Delivery
Source: Nutrients. 2020 Jun 15;12(6):1779. doi: 10.3390/nu12061779 (PMC7353435; doi:10.3390/nu12061779)
Supplement: Supplementary file 1 [file nutrients-12-01779-s001.pdf]

Table S1. Characteristics of population participant in the post-partum weight retention study (n=167)

	Weight retention study (n=167)
Weight gain (Kg)	12 [10-15]
Low	58 (34.7)
Recommended	73 (43.7)
High	36 (21.6)
Pre-gestational BMI (Kg/m^2)	22.31 [20.44-24.46]
NP	118 (70.65)
OW	38 (22.8)
LW	11 (6.6)
Antibiotic pregnancy (positive cases)	57 (33.9)
Gestational age (weeks)	40 [39-40]
Delivery mode	
Vaginal	111 (66.5)
Emergency C-section	25 (14.9)
Elective C-section	31 (18.5)
Anthropometric data (maternal weight)	
Pregestational (Kg)	60.50 [54.5-66]
Delivery (Kg)	72 [67.5-80]
7 days (Kg)	67.50 [61-73.88]
15 days (Kg)	65.7 [60-71.8]
31 days (Kg)	64 [58-70.5]
2 months (Kg)	63 [57.50-70.13]
4 months (Kg)	61.50 [56-69]

Normally distributed data was presented as mean ± SD and non-normal data as median [IQR]. Categorical variables were expressed as positive cases (percentage). In the case of microbiota study population. only a subset of participants had anthropometric data during post-partum period (n=40).

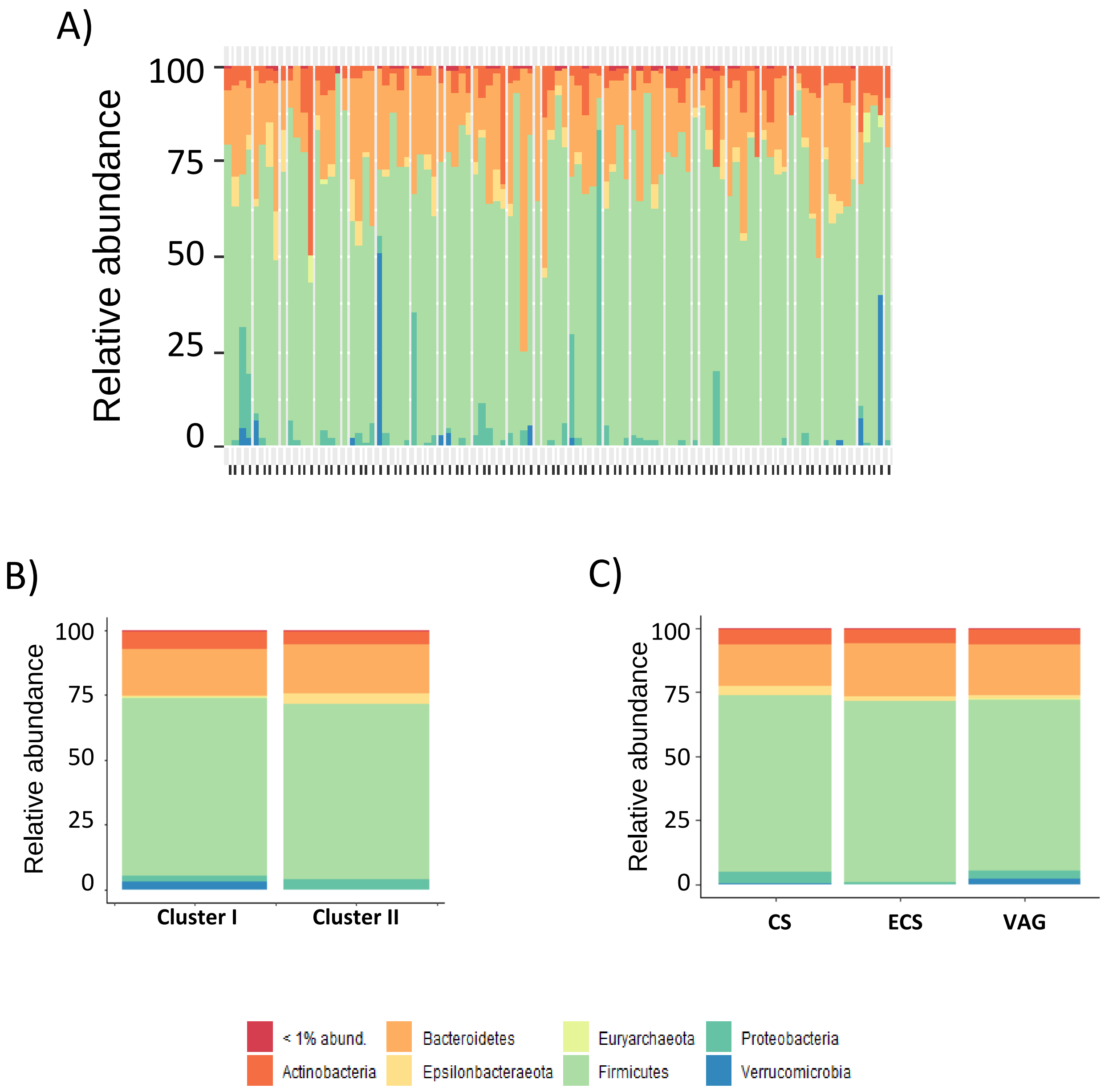

Figure S1. Maternal microbiota composition at phylum level at delivery time. Bar plot at phylum level of maternal microbiota according to individual (**A**). cluster (**B**) and delivery mode (**C**).

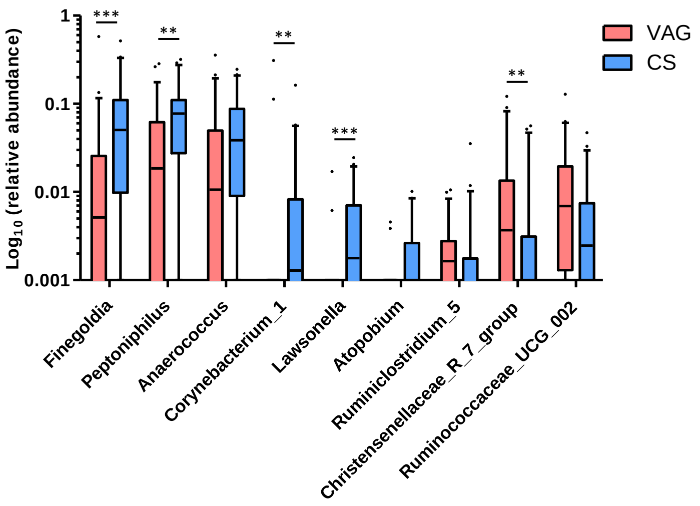

Figure S2. Differences in maternal microbiota at delivery time according to delivery mode. Boxplot of the main genera that marked the difference of maternal microbiota composition between delivery mode grouping both C-section modes. Data was transformed by log10 of relative abundance of each genus for plotting. Whiskers represented 5-95 percentile interval. * $p<0.05$, ** $p<0.01$, *** $p<0.001$.

Table S2. Occurrence of genera from maternal microbial core at delivery according to delivery mode.

Taxa	CS (%)	ECS (%)	VAG (%)
UBA1819	54	56	77
Subdoligranulum	91	100	89
Streptococcus	91	100	91
S5A14a	57	67	51
Ruminococcus_2	57	44	62
Ruminococcaceae_UCG014	43	67	60
Ruminococcaceae_UCG013	63	78	77
Ruminococcaceae_UCG005	66	67	77
Ruminococcaceae_UCG004	57	56	57
Ruminococcaceae_UCG003	40	56	60
Ruminococcaceae_UCG002	69	78	89
Ruminococcaceae_NK4A214_group	57	56	62
Ruminiclostridium_9	60	78	72
Ruminiclostridium_5	86	78	89
Roseburia	63	89	81
Romboutsia	71	78	77
Prevotella_6	40	67	60
Prevotella	86	100	79
Porphyromonas	83	89	77
Peptoniphilus	91	89	91
Parasutterella	40	67	55
Parabacteroides	77	89	89
Oscillibacter	66	89	74
Odoribacter	71	78	79
Murdochiella	69	78	57
Mobiluncus	74	89	62
Lawsonella	89	89	60
Lactobacillus	71	78	55
Lachnospiraceae_NK4A136_group	66	78	87
Lachnospiraceae_ND3007_group	63	67	66
Lachnospiraceae_FCS020_group	57	44	70
Lachnospira	69	89	77
Lachnoclostridium	77	89	87
Intestinibacter	51	89	58
Fusicatenibacter	69	89	81
Flavonifractor	43	78	58
Finegoldia	94	100	83
Family_XIII_AD3011_group	46	78	72
Faecalibacterium	86	78	96
Ezakiella	83	89	89
EscherichiaShigella	83	100	98
Erysipelotrichaceae_UCG003	69	67	74
Dorea	94	89	89
Dialister	83	100	75
Corynebacterium_1	74	78	42
Coprococcus_3	80	56	79
Coprococcus_1	74	67	72
Collinsella	83	78	81
Clostridium_sensu_stricto_1	49	56	68
Christensenellaceae_R7_group	60	67	92
Campylobacter	89	78	75
Butyricicoccus	69	89	75
Blautia	97	89	94
Bilophila	49	56	66
Bifidobacterium	94	100	89
Bacteroides	97	100	98
Anaerostipes	77	89	81
Anaerococcus	91	100	85
Alistipes	83	89	94
Agathobacter	91	78	81

Occurrence was expressed as percentage of samples with presence of each genera within a delivery mode group. VA (vaginal delivery), ECS (emergency C-section), CS (elective C-section).
